# Efficacy and safety of QL0911 in adult patients with chronic primary immune thrombocytopenia: A multicenter, randomized, double-blind, placebo-controlled, phase III trial

**DOI:** 10.2478/jtim-2023-0106

**Published:** 2023-12-20

**Authors:** Hu Zhou, Shouqing Han, Jie Jin, Ruibin Huang, Xinhong Guo, Xuliang Shen, Binghua Wang, Xin Wang, Hongxia Yao, Xin Du, Meijuan Huang, Xuehong Ran, Wei Wang, Tonghua Yang, Feng Zhang, Changcheng Zheng, Xuelan Zuo, Rong Fu, Da Gao, Zheng Ge, Ying Han, Yujie Li, Xiaoyan Kang, Yan Shi, Ming Hou

**Affiliations:** Department of Hematology, Henan Cancer Hospital, Affiliated Cancer Hospital of Zhengzhou University, Zhengzhou 450008, Henan Province, China; Department of Hematology, Qilu Hospital, Cheeloo College of Medicine, Shandong University, Jinan 250012, Shandong Province, China; Department of Hematology, The First Affiliated Hospital Zhejiang University School of Medicine, Hangzhou, 310003, Zhejiang Province, China; Department of Hematology, The First Affiliated Hospital of Nanchang University, Nanchang 330006, Jiangxi Province, China; Department of Hematology, The First Affiliated Hospital of Xinjiang Medical University, Urumqi 830054, Xinjiang Uygur Autonomous Region, China; Department of Hematology, Heping Hospital Affiliated to Changzhi Medical College, Changzhi 046000, Shanxi Province, China; Department of Hematology, Weihai Central Hospital, Weihai 264400, Shandong Province, China; Department of Hematology, Suining Central Hospital, Suining 629099, Sichuan Province, China; Department of Hematology, Hainan General Hospital, Haikou 570311, Hainan Province, China; Department of Hematology, Shenzhen Second People’s Hospital, Shenzhen 518035, Guangdong Province, China; Department of Hematology, Fujian Medical University Union Hospital, Fuzhou 350001, Fujian Province, China; Department of Hematology, Weifang People’s Hospital, Weifang 261044, Shandong Province, China; Department of Hematology, The Affiliated Hospital of Qingdao University, Qingdao 266000 Qingdao, Shandong Province, China; Department of Hematology, The First People’s Hospital of Yunnan Province, Kunming 650031, Yunnan Province, China; Department of Hematology, The First Affiliated Hospital of Bengbu Medical College, Bengbu 233004, Anhui Province, China; Department of Hematology, Anhui Province Hospital, Hefei 230002, Anhui Province, China; Department of Hematology, Zhongnan Hospital of Wuhan University, Wuhan 430071, Hubei Province, China; Department of Hematology, Tianjin Medical University General Hospital, Tianjin 300052, China; Department of Hematology, The Affiliated Hospital of Inner Mongolia Medical University, Tongliao 028000, Inner Mongolia, China; Department of Hematology, Zhongda Hospital Southeast University, Nanjing 210009, Jiangsu Province, China; Department of Medicine, Qilu Pharmaceutical Co., Ltd, Jinan 250100, Shandong Province, China; Statistics and Statistical Programming, Qilu Pharmaceutical Co., Ltd, Jinan 250100, Shandong Province, China; Department of Hematology, Qilu Hospital of Shandong University, Jinan 250012, Shandong Province, China; Department of Hematology, Shandong Provincial Key Laboratory of Immunohematology, Qilu Hospital of Shandong University, Jinan 250012, Shandong Province, China

**Keywords:** primary immune thrombocytopenia, thrombopoietin receptor agonist, biosimilar, platelet response, efficacy, safety

## Abstract

**Objective:**

QL0911, a recombinant human thrombopoietin mimetic peptide-Fc fusion protein, is a romiplostim (Nplate^®^) biosimilar used to treat primary immune thrombocytopenia (ITP). This phase III study aimed to assess the efficacy and safety of QL0911 in adult patients with chronic primary ITP over a 24-week treatment period.

**Methods:**

We conducted a double-blind, placebo-controlled, phase III study in patients diagnosed with primary ITP for at least 12 months who had received at least one first-line ITP treatment with no response or recurrence after treatment, or who relapsed after splenectomy at 44 sites in China. Patients were randomly allocated (2:1 ratio) to QL0911 or placebo injection subcutaneously once weekly at an initial dose of 1 μg/kg for 24 weeks. The doses were adjusted to maintain the target platelet counts from 50 × 109/L to 200 × 109/L. Patients and investigators were blinded to the assignment. The primary endpoints were the proportion of patients who achieved a durable platelet response at week 24 (platelet count, ≥ 50 × 109/L during 6 of the last 8 weeks of treatment) and safety. The study was registered at ClinicalTrials.gov (NCT05621330).

**Results:**

Between October 2019 and December 2021, 216 patients were randomly assigned (QL0911,144; placebo,72). A durable platelet response was achieved by significantly more patients in the QL0911 group (61.8%, 95% CI: 53.3-69.8; *P* < 0.0001) than in the placebo group (0%). The mean duration of platelet responses was 15.9 (SE: 0.43) weeks with QL0911, and 1.9 (SE:0.26) week with placebo. Consistent results were achieved in subgroup analyses categorized by baseline splenectomy status (yes/no), concomitant ITP treatment (yes/no), and baseline platelet count (≤ 10 × 109/L, > 10 × 109/L, ≤ 20 × 109/L, > 20 × 109/L, and < 30 × 109/L). The incidence of TEAEs was comparable between the QL0911 and the placebo groups (91.7% and 88.9%, respectively). The most common adverse events overall were ecchymosis (28.5% for QL0911 *vs*. 37.5% for placebo), upper respiratory tract infections respiratory tract infections (31.9% for QL0911 *vs*. 27.8% for placebo), and gingival bleeding (17.4% for QL0911 *vs*. 26.4% for placebo).

**Conclusion:**

QL0911 was well-tolerated and increased and maintained platelet counts in adults with ITP. QL0911, a biosimilar to romiplostim (Nplate®), may be a novel treatment option for patients with ITP who have failed or relapsed from first-line treatment in China. Ongoing studies will provide further data on long-term efficacy and safety in such patient populations.

## Introduction

Primary immune thrombocytopenia (ITP), an acquired autoimmune hemorrhagic disorder, is characterized by an isolated reduction in the peripheral blood platelet count without a clear cause.^[1]^ ITP in adults displays more chronic features; therefore, patients may have a higher risk of bleeding, which seriously affects their quality of life (QoL), and in some cases, is worse than that in cancer patients.^[2]^ Both the international consensus report on the investigation and management of primary ITP^[3]^ and the Chinese guidelines on the diagnosis and management of adult primary ITP (version 2020)^[4]^ indicate that the treatment goals for ITP are to maintain a safe platelet count, reduce bleeding events, and improve QoL by minimizing adverse reactions/treatment-related toxicities. Current first-line treatment regimens for chronic ITP include corticosteroids or intravenous high-dose immunoglobulins (IVIg) in certain cases to treat patients with a high bleeding risk.^[3, 4, 5]^ However, both corticosteroids and IVIg are associated with transient efficacy and obvious toxicity, and relapse after discontinuation is common.[6] In addition, evidence suggests that approximately 30% of patients with ITP treated with corticosteroids do not achieve an initial platelet response, and more than 95% report adverse effects such as hypertension, hyperglycemia, and acute gastric mucosal lesions, and some patients with long-term use of corticosteroids may develop osteoporosis or femoral head necrosis.[7,8] The choice of treatment for patients with ITP requires careful weighing of the benefits and potential risks of these treatment options.

Our understanding of the pathophysiology and therapeutic approaches for ITP has deepened significantly over the last few decades. Thrombopoietin receptor agonists (TPO-RAs) are a novel class of ITP therapeutics that not only promote the production of platelets from existing megakaryocytes but also stimulate megakaryocytosis in the bone marrow, resulting in an increase in platelet count.[9-11] ITP-related national and international guidelines explicitly recommend TPO-RAs as key options for second-line treatment.[3-5] Currently, five TPO-RAs, including eltrombopag, avatrombopag, heterotrombopag, recombinant human thrombopoietin (rhTPO), and romiplostim.[12,13] Among them, romiplostim (Nplate^®^), a recombinant thrombopoiesis-stimulating Fc-peptide fusion protein, was the first US Food and Drug Administration (FDA)-approved second-generation TPO-RA for the treatment of chronic ITP in adults^[14]^ and was approved in China in January 2022. A substantial number of clinical trial results have confirmed that romiplostim administered once weekly through subcutaneous injection induces a durable platelet response with a rapid onset of action and minimal adverse events in adult ITP patients.^[15, 16, 17, 18, 19, 20]^

QL0911, a recombinant human thrombopoietin-mimetic peptide-Fc fusion protein for injection, is a biosimilar to romiplostim (Nplate^®^) developed by Qilu Pharmaceutical Co., Ltd. for the treatment of chronic ITP in adults and other indications. Preclinical studies of QL0911 revealed that it was highly consistent with licensed romiplostim in terms of structure, physicochemical properties, biological activity, and pharmacokinetics (PK), and that it was well tolerated without severe drug-related toxicity (data submitted for publication). A randomized, double-blind, placebo-controlled, dose-escalation, phase I clinical trial in healthy volunteers demonstrated the safety and tolerability of a single subcutaneous injection of QL0911 at a dose of 0.3–2 μg/kg (Table S1 and Figure S1). The dosage regimen in another Ib study was designed as dose escalation (1, 3, and 6 μg/kg), continuous treatment (up to 12 weeks at adjustable doses, up to 10 μg/kg), and treatment expansion (up to 24 weeks at adjustable doses, up to 10 μg/kg) phase. Preliminary results showed that multiple doses of QL0911 were safe and well-tolerated in patients with ITP, and 72.0% (18/25) of the patients had at least one platelet response after receiving QL0911 (data submitted for publication). The dose of QL0911 can be adjusted according to platelet count, and the initial dose (1 μg/kg) and maximum dose (10 μg/kg) also provide a reference for phase III clinical trials.

Therefore, we conducted this phase III study to demonstrate the superiority of QL0911 over placebo in raising and maintaining platelet counts in patients with chronic ITP within a target range (≥ 50 × 109/L) over a 24-week treatment period and to evaluate the efficacy and safety of long-term treatment over 42 weeks.

## Methods

### Study design

This multicenter phase III study (NCT05621330) of QL0911 in adult patients with chronic ITP was conducted at 44 sites (Table S2) across China from October 2019 to December 2021. This study consisted of a randomized, double-blind, placebo-controlled 26-week treatment period, sequentially followed by an open-label, single-arm 12-week treatment period and an additional 4-week safety follow-up period. All patients who entered the open-label period received the investigational drug, QL0911. This study was conducted in strict compliance with the Declaration of Helsinki, International Council for Harmonization of Technical Requirements for Pharmaceuticals for Human Use (ICH) for Good Clinical Practice (GCP), and appropriate local regulatory requirements. The study protocol, informed consent form, and related amendments were approved by the ethics committee at each site. Written informed consent was obtained from all the patients.

### Patients

The study population included patients diagnosed with primary ITP for at least 12 months, who had received at least one first-line ITP treatment with no response or recurrence after treatment and who had a poor response or relapse after splenectomy. Eligible patients were ≥18 years of age and had a platelet count < 30 × 109/L within 48 h before the first dose. The patients had an Eastern Cooperative Oncology Group (ECOG) performance status of 0–2. Patients were excluded if they (1) had a history of bone marrow stem cell abnormalities or myelodysplastic syndrome, other than ITP-specific changes; (2) arterial thrombosis or venous thromboembolism, severe cardiovascular diseases, malignant tumors, and secondary thrombocytopenia caused by autoimmune diseases*; (*3) underwent splenectomy within 12 weeks before the first dose; (4) had received ITP treatments (including rescue treatment) within 2 weeks before the first dose; (5) had received romiplostim (Nplate^®^) or eltrombopag (Revolade^®^), rhTPO or other agents that stimulate TPO receptors (also known as c-Mpl), and hematopoietic growth factors (HGFs) within 4 weeks before the first dose; (6) had received antineoplastic agents within 8 weeks before the first administration; however, when treating ITP with hypomethylating agents (HMA) such as decitabine, a 4-week washout period was acceptable, as judged by the investigator; (7) had received antibody-based therapies within 14 weeks before the first dose; (8) had serum creatinine or total bilirubin > *1.5* upper limit of normal (ULN), alanine transaminase (ALT) or aspartate transaminase (AST) > 3 ULN, hemoglobin < 100 g/L, absolute neutrophil count <1.5×109/L; and (9) had prothrombin time (PT) or prothrombin time-international normalized ratio (PT-INR) or activated partial thromboplastin time (APTT) exceeded 20% of the reference range of normal values.

### Randomization and masking

In the randomized, double-blind, placebo-controlled phase, patients were randomly assigned in a 2:1 ratio to either the QL0911 or placebo treatment group. The placebo was an excipient without the active ingredient QL0911 and was not distinguishable in appearance or shape. Block randomization was performed with a block size of six and stratified according to previous splenectomy status (yes/no) to ensure balance between the groups. Patients and personnel at all investigational sites (including investigators and assessors) involved in this trial were blinded to the treatment assignments. Key data were kept confidential at weeks 25 and 26 until unblinding before week 27. Emergency unblinding can be conducted in response to emergency situations (medical emergencies or serious medical conditions). Patients in the placebo group entered the open-label treatment period after unblinding and received QL0911 treatment.

### Treatment interventions

During the double-blind treatment period, QL0911 or the placebo was initially injected subcutaneously once a week at a dose of 1 μg/kg and titrated weekly in 1 μg/kg increments until a maximum dose of 10 μg/kg or a target platelet count of 50 × 109/L was reached. Dose modifications were made based on the individual platelet responses. If the platelet count increased to 50 × 109/L–200 × 109/L, the administered dose was maintained. If the platelet count remained between 200 × 109/L and 400 × 109/L for 2 weeks, consecutively the dose was reduced by 1 μg/kg on the next scheduled dosing day. If the platelet counts were 400 × 109/L or greater, dosing was withheld until platelet counts decreased to below 200 × 109/L, after which dosing was reduced by 1 μg/kg. Dosing was withheld when the platelet count exceeded 400 × 109/L after receiving rescue treatments until the platelet count decreased to below 200 × 109/L, and dosing was resumed at the last dosage.

### Study endpoints assessment

The patients were assessed for efficacy during the double-blind and open-label treatment periods. Treatment response was defined as a platelet count of ≥ 50 × 109/L. The primary endpoint was the proportion of patients who achieved a durable platelet response at week 24 of the double-blind treatment period. A durable platelet response was defined as a platelet count ≥ 50 × 109/L during six of the last eight weeks of treatment. Secondary endpoints included: the proportion of patients with weekly platelet responses within 24 weeks of treatment; the proportion of patients who achieved platelet count ≥ 30 × 109/L at least a two-fold increase from baseline platelet count without bleeding during the 24-week double-blind period; the time required from the first dosing to the first platelet response within 24 weeks of treatment; mean weeks of platelet response within 24 weeks of treatment; the proportion of patients who had received rescue treatment within 24 weeks of treatment; mean maximum weeks of platelet response within 24 weeks of treatment; the platelet counts at every scheduled visit, and the proportion of patients with platelet responses at each week.

Safety was assessed by monitoring adverse events (AEs) and serious adverse events (SAEs) (graded with the Common Terminology Criteria for Adverse Events/CTCAE, version 5.0). AEs and treatment-emergent adverse events (TEAEs) were coded according to Medical Dictionary for Regulatory Activities version 24.1 (MedDRA).

### Determination of sample size

Based on safety considerations, if the sample size was 120 in the experimental group and the actual incidence of specific AEs in patients receiving the investigational drug was 2%, the probability of at least one event being observed in the 120 patients was 91.1%. At the end of the study, if no AE occurred in the 120 treated patients, the actual incidence of AE was < 1.34% with 80% confidence and 1.91% with 90% confidence. If 5 AEs were observed in the 120 treated patients, the actual incidence of AE was at least 2.6% with 80% confidence (at least 2.0% with 90% confidence). With an experimental group to placebo group ratio of 2:1 and a dropout rate of approximately 15%, 216 patients were enrolled (QL0911:144; placebo:72).

### Statistical analyses

All eligible randomized patients who received at least one dose of the investigational drug were included in the full analysis set (FAS). All patients who received at least one dose of the investigational drug and for whom safety data were available were included in the safety analysis set (SS). The per-protocol set (PPS) was defined as all patients who met the FAS without major protocol deviations that were judged to have a significant impact on the outcomes.

The incidence of a durable platelet response during the double-blind 24-week treatment period was compared between the two groups using the Cochran-Mantel-Haenszel test stratified by splenectomy. For secondary endpoints, the differences between the two groups for dichotomous endpoints were analyzed using the Cochran-Mantel-Haenszel test, stratified according to whether a previous splenectomy had been performed. Patients who received rescue treatments for increasing platelet count were not considered to have a platelet response within 4 weeks after initiation of rescue treatments. Patients who discontinued treatment early in the study were not considered to have a weekly platelet response after discontinuation. Continuous endpoints were analyzed using the rank-sum test. The time from the first dose to the first platelet count of ≥ 50 × 109/L during the double-blind 24-week treatment period was compared between the groups using the log-rank test.

## Results

### Patients

**Figure 1:**
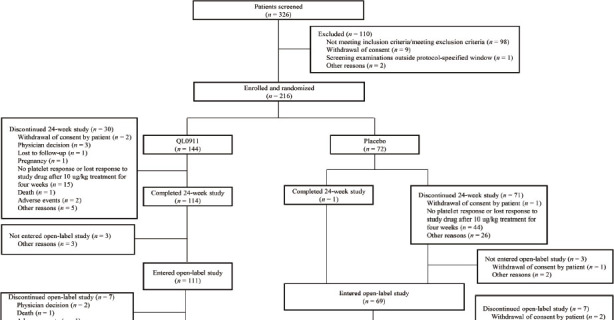
Patient disposition.

Between October 18, 2019, and December 13, 2021, a total of 326 patients were screened, of whom 216 were eligible and randomly assigned to the QL0911 group (*n* = 144) and the placebo group (*n* = 72) (Figure 1). One hundred and fourteen patients (79.2%) in the QL0911 group and 1 (1.4%) in the placebo group completed the 24-week double-blind treatment period. The main reason for discontinuation of the study was the absence of a platelet response or loss of response to QL0911 after 4 weeks of treatment with 10 μg/kg (QL0911:10.4%, *n* = 15; placebo:61.1%,*n* = 44). Of the 69 patients in the placebo group who entered the open-label phase, 62 were treated with QL0911 and 7 discontinued the open-label study.

The baseline demographic and disease characteristics of the eligible population in the QL0911 and placebo groups are shown in Table 1 and are generally comparable. Most patients in the QL0911 and placebo groups were female (75.0% and 59.7%, respectively). Mean baseline platelet counts (± SD) were (13.07 ± 8.58) × 109/L in the QL0911 group and (13.15 ± 8.31) × 109/L in the placebo group, respectively. The median duration from initial ITP diagnosis to study enrollment in the QL0911 and placebo groups was 4.91 (range: 0.99–51.59) and 4.15 (range: 1.00–29.62) years. In both groups, the majority of patients had not undergone prior splenectomy (QL0911 *vs*. placebo, 91% *vs*. 90.3%).

**Table 1:** Baseline demographic and disease characteristics of enrolled patients (FAS)

	QL0911 (*n* = 144)	Placebo (*n* = 72)
Age, years, median (range) Sex, *n* (%)	42.0 (18–75)	48.5 (18–83)
Female	108 (75.0)	43 (59.7)
Weight (kg), mean (SD)	62.55 (11.63)	66.04 (11.53)
ECOG PS, *n* (%)		
0	90 (62.5)	39 (54.2)
1	52 (36.1)	32 (44.4)
2	2 (1.4)	1 (1.4)
Baseline 109/L), mean platelet (SD) count (×	13.07 (8.58)	13.15 (8.31)
Time from initial ITP diagnosis to ICF date, years, median (range) Prior splenectomy, *n* (%)	4.91 (0.99–51.59)	4.15 (1.00–29.62)
Yes	13 (9.0)	7 (9.7)
No	131 (91.0)	65 (90.3)
Concomitant ITP treatment, *n* (%)	57 (39.6)	43 (59.7)
Prior first-line ITP treatment, *n* (%)	144 (100.0)	72 (100.0)

ECOG: Eastern Cooperative Oncology Group; ICF: informed consent form; PS: performance status; SD: standard deviation.

### Dosage exposure

**Figure 2:**
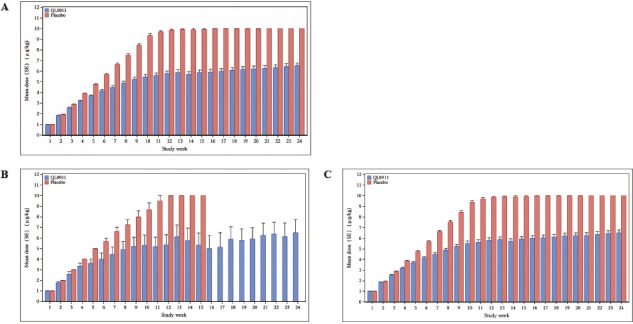
The mean doses of QL0911 or placebo administered weekly to patients during the double-blind, 24-week treatment period in the total population (A), splenectomized (B), and non-splenectomized patients (C).

The mean doses of QL0911 or placebo administered to patients each week during the 24-week double-blind treatment period are shown in Figure 2. The mean administered dose of QL0911 continuously increased from the initial dose of 1–5.8 μg/kg for the first 12 weeks, compared with 9.9 μg/kg for those administered placebo. Patients who received QL0911 had a mean exposure dose (± SD) of 5.43 ± 2.5 μg/kg for a durable platelet response. No persistent platelet response was observed in the placebo group.

### Efficacy

Two hundred sixteen and 211 randomized patients were included in the FAS and PPS groups, respectively. The proportions of patients achieving durable platelet responses for the FAS and PPS during the double-blind 24-week treatment period are shown in Table 2. Approximately 61.8% (95%CI 53.3–69.8%) of patients achieved durable platelet responses after 24 weeks of QL0911 treatment for FAS compared with none of the patients in the placebo group (*P* < 0.0001). The rate difference between two groups was 61.8% (95% CI, 53.3–69.8%) (*P* < 0.0001). In the analysis of PPS, the durable platelet response rate was significantly higher in QL0911-treated patients compared with those receiving placebo (62.6% *vs*. 0.0%), with the rate difference being 62.6% (95% Cl, 54.0–70.6%) (*P* < 0.0001).

**Table 2:** Incidence of durable platelet response during the double-blinded, 24-week treatment period

	QL0911 (*n* = 144/139)		Placebo (*n* = 72)		QL0911 *vs*. Placebo		
	*n* (%)	95% CI^*^	*n* (%)	95% CI	Rate difference (unadjusted)	95% CI^**^	*P*
FAS (*n* = 216)	89 (61.8)	(53.3, 69.8)	0 (0)	(0.0, 5.0)	61.80	(53.3, 69.8)	＜ 0.0001
PPS (*n* = 211)	87 (62.6)	(54.0, 70.6)	0 (0)	(0.0, 5.0)	62.60	(54.0, 70.6)	＜ 0.0001

FAS: full analysis set; PPS: per-protocol set. ^*^95% CI was estimated using the Clopper and Pearson method. ^**^95% CI was estimated using the Santner-Snell exact method. *P*-values were calculated using the two-sided Fisher’s exact test.

**Figure 3:**
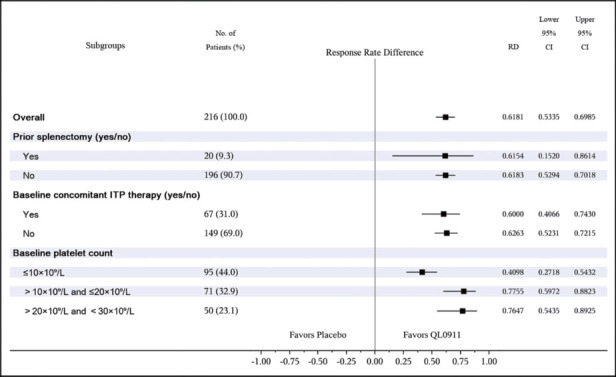
Incidence of durable platelet response in subgroups during the double-blind, 24-week treatment period (FAS).

The results of the subgroup analyses based on the primary efficacy endpoint are shown in a forest plot (Figure 3). All patients included in the FAS were analyzed by subgroup, according to splenectomy status (yes/no), concomitant therapy (yes/no), or baseline platelet counts (≤ 10 × 109/L, > 10 × 109/L and ≤ 20 × 109/L, > 20 × 109/L, and < 30 × 109/L). The trend in the results of all subgroup analyses in patients treated with QL0911 or the placebo was consistent with the FAS analysis. In each of the predefined subgroups, the proportion of patients with durable platelet responses after receiving QL0911 was higher than that after receiving placebo.

**Figure 4:**
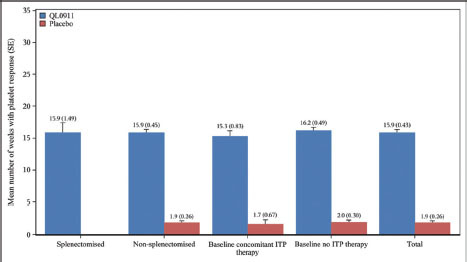
Number of weeks to achieve platelet response in the total patient population and subgroups during the double-blind, 24-week treatment period.

The mean number of weeks maintaining a platelet response was much longer with QL0911 than with placebo for the FAS (15.9 [SE:0.43] weeks vs. 1.9 [SE:0.26] weeks). The results of all subgroup analyses were consistent with the trends in the FAS (Figure 4).

**Figure 5:**
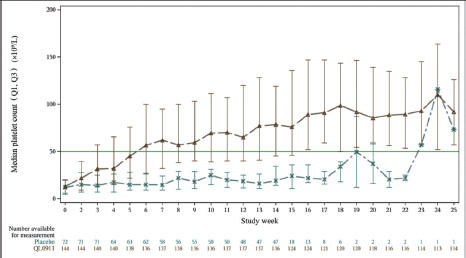
Median platelet counts of the FAS at each visit during the double-blind, 24-week treatment period.

The median platelet counts of the FAS at each visit during the double-blind 24-week treatment period are shown in Figure 5. The median platelet count of patients in the QL0911 group was ≥ 50 × 109/L after 6 weeks of treatment, and the platelet count continued to increase with treatment duration. The median platelet count in the placebo group fluctuated predominantly around the baseline. Most patients discontinued the double-blind phase of the study because they did not respond to treatment. Platelet counts at each weekly visit during the 24-week treatment period by subgroup are presented in Figure S2.

Platelet response rate and proportion of patients with weekly platelet count ≥ 30 × 109/L and with a 2-fold increased platelet count from baseline and without bleeding events are presented in Figure S3 and S4.

**Figure 6:**
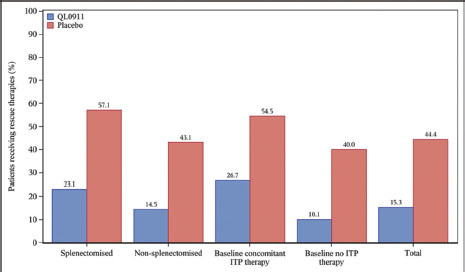
Proportion of patients receiving rescue therapies during the double-blind, 24-week treatment period.

As indicated in Figure 6, rescue therapies were administered to 22 (15.3%) patients receiving QL0911 versus 32 (44.4%) patients receiving placebo (rate difference: -29.1, 95% CI -42.0 to -16.2; *P* < 0.0001). The proportion of patients who received rescue treatment was higher in the placebo group than in the QL0911 group. Furthermore, Kaplan–Meier curve of time to first achieving platelet count ≥ 50 × 109/L is shown in Figure S5. The median time to the first platelet response in the QL0911 group was significantly shorter than patients in the placebo (3.29 *vs*. 18.86 weeks, *P* < 0.0001). Subgroup analyses of the patients showed a similar trend in terms of time to the first platelet response.

### Safety

A safety summary of the 24-week double-blind treatment is presented in Table 3. A total of 132 (91.7%) patients in the QL0911 group and 64 (88.9%) in the control group reported TEAEs during the 24-week double-blind treatment period. In the QL0911 group, the most frequent TEAE was upper respiratory tract infection (31.9%), followed by ecchymosis (28.5%) and urinary tract infection (19.4%). The most common TEAE in the placebo group was ecchymosis (37.5%). Grade ≥ 3 TEAEs were reported in 35 (24.3%) patients in the QL0911 group, and 31 (43.1%) patients in the placebo group, respectively. In both groups, the most common grade ≥ 3 TEAE was decreased platelet count (QL0911 *vs*. placebo, 12.5% *vs*. 27.8%).

**Table 3:** Adverse events (≥10% in total population) within 24-week treatment period (SS)

	QL0911	Placebo	Total
	(*n* = 144)	(*n* = 72)	(*n* = 216)
Any TEAE, *n* (%)	132 (91.7)	64 (88.9)	196 (90.7)
Ecchymosis	41 (28.5)	27 (37.5)	68 (31.5)
Upper respiratory tract infection	46 (31.9)	20 (27.8)	66 (30.6)
Gingival bleeding	25 (17.4)	19 (26.4)	44 (20.4)
Petechiae	25 (17.4)	18 (25.0)	43 (19.9)
Platelet count decreased	18 (12.5)	20 (27.8)	38 (17.6)
Urinary tract infection	28 (19.4)	9 (12.5)	37 (17.1)
Anemia	17 (11.8)	13 (18.1)	30 (13.9)
Arthralgia	19 (13.2)	5 (6.9)	24 (11.1)
Headache	14 (9.7)	9 (12.5)	23 (10.6)

TETA: treatment-emergent adverse event.

Serious adverse events (SAEs) were reported in 25 (17.4%) and 22 (30.6%) patients in the QL0911 and placebo groups, respectively. Treatment-related SAEs occurred in one patient in the QL0911 group and one patient in the placebo group. One patient assigned to QL0911 reported treatment-related SAE (myocarditis). The patient recovered after the study drug was discontinued and received proper medication.

Three patients assigned to QL0911 died during the study: one from cerebral hemorrhage and brain herniation, one from cerebral hemorrhage and gastrointestinal bleeding, and one from a decreased platelet count and cerebral hemorrhage (cerebral hemorrhage occurred after the 24-week treatment period). Three deaths were determined to be unrelated to QL0911 by the investigator.

No thromboses or malignant neoplasms were observed. Four patients in the QL0911 group had bone marrow reticulin fibrosis. All events were grade 1.

None of the patients assigned to the QL0911 group tested positive for antibodies against QL0911. One patient tested positive for antibodies against thrombopoietin at baseline, and the result was negative after week 13.

## Discussion

QL0911, a romiplostim (Nplate^®^) biosimilar developed in China for the treatment of chronic primary ITP in adults, has demonstrated safety and PK/PD in phase I and Ib clinical trials. The results of this phase III study confirm its safety and efficacy. A durable platelet response was achieved in 89 of the 144 (61.8%) patients treated with QL0911. QL0911 was significantly superior to the placebo at an initial dose of 1 μg/kg once weekly for all primary and secondary efficacy endpoints during the double-blind 24-week treatment period.

Splenectomy is the second-line treatment for ITP with a high response rate. In clinical practice, splenectomy rates declined with the availability of rituximab and TPO-RAs.^[21]^ Consistent with this trend, the proportion of patients who had undergone prior splenectomy was low in this study (9.3%). Although a statistical test was not performed for the subgroups in patients with or without prior splenectomy, QL0911 showed consistent superiority to the placebo in inducing a durable platelet response. Moreover, no difference in the rate of durable platelet response was observed between patients with splenectomized and nonsplenectomized ITP assigned to QL0911.

To our knowledge, this is the first study to report the efficacy of a romiplostim (Nplate^®^) biosimilar in Chinese patients with chronic ITP. Although direct comparison with romiplostim (Nplate^®^) was not included in this study, the primary efficacy endpoint, as well as secondary efficacy endpoints used in this study are the same as that used in the phase III study of romiplostim (Nplate^®^). In patients without a previous splenectomy, 61.8% of the QL0911 group achieved durable platelet responses, which were numerically similar to those reported for romiplostim (Nplate^®^). The overall response rate, which was less strictly defined in non-splenectomized patients (84%), was comparable to that of romiplostim (Nplate^®^).[15] The same trend was observed for another secondary efficacy endpoint, including the number of weeks with platelet response, platelet count at each visit, and the proportion of patients receiving rescue therapies. Therefore, QL0911 demonstrated a trend toward clinical equivalence with romiplostim (Nplate^®^) in a Chinese patient population with chronic ITP.

Safety data from the present study demonstrated that QL0911 was well tolerated and no new safety signals were identified. The overall incidence of TEAEs was comparable between the QL0911 and placebo groups (91.7% and 88.9%, respectively). Most events were mild-to-moderate and appeared to be related to the underlying disease. For patients assigned to QL0911, the incidence of severe bleeding events (grade ≥ 3) was low (0%–2%). Thromboembolic events were not observed during QL0911 treatment, which was also uncommon in patients receiving romiplostim (Nplate^®^).[15]

In patients with ITP, the goal of treatment is to increase the platelet count to a safe level by minimizing bleeding. However, in clinical practice, patients with ITP do not always respond to first-line treatment with corticosteroids or IVIg. Currently, among the five TPO receptor agonists for the treatment of ITP that have been marketed worldwide, romiplostim, the only peptide TPO-RA, has a peptide domain that binds to the TPO receptor and stimulates platelet production, and the carrier antibody Fc domain can prolong the circulation half-life.[1,14,22] Romiplostim (Nplate^®^), a long-acting TPO-RA requiring once-weekly dosing, was approved in China in January 2022. The rise in biosimilars offers exciting opportunities to improve healthcare sustainability and allows patients to benefit from more treatment options.[23] Owing to the superior efficacy and safety of romiplostim in ITP and considering the limited treatment options, there is more interest in Nplate^®^ biosimilar, although there are still multiple challenges in the development and adoption of biosimilars.[24,25] To the best of our knowledge, QL0911 is the first Nplate^®^ biosimilar in China to undergo a phase III clinical study.

In conclusion, the results of this phase III trial confirmed that QL0911 at a starting dose of 1 μg/kg once weekly was superior to placebo in increasing platelet counts and maintaining a durable platelet response during a 24-week double-blind treatment period in adults with ITP and was well tolerated. QL0911 as a biosimilar of romiplostim (Nplate^®^) may be a new treatment option for ITP patients with failure or relapse from first-line treatment in China.

## Supplementary Material

Supplementary material
