# Supplementary material for: Efficacy and safety of QL0911 in adult patients with chronic primary immune thrombocytopenia: A multicenter, randomized, double-blind, placebo-controlled, phase III trial
Source: J Transl Int Med. 2023 Dec 20;11(4):423–32. doi: 10.2478/jtim-2023-0106 (PMC10732573; doi:10.2478/jtim-2023-0106)
Supplement: Supplementary file 1 — Supplementary material [file jtim-2023-0106_sm.pdf]

Supplementary Materials

Table S1: Pharmacodynamic parameters after a single dose of QL0911 or placebo in phase I study

	QL0911 (μg/kg)			Placebo (*n*= 6)
	0.3 (*n* = 8)	1 (*n* = 8)	2 (*n* = 8)	
Tmax, days, median (range)	13.50 (10-43)	11.00 (10-14)	12.50 (12-17)	25.50 (13-43)
P0 (10^9/L), mean (SD)	243.63 (34.50)	220.25 (31.10)	225.13 (55.31)	206.00 (33.46)
P_{max}(10^9/L), mean (SD)	301.75 (51.88)	358.25 (55.72)	373.38 (70.02)	250.00 (41.67)
P_{max}/P0(%), mean (SD)	123.67 (8.74)	163.53 (18.40)	171.12 (40.67)	121.67 (11.39)
AUC_{0-43d}(d*10^9/L), mean (SD)	11174.57 (1515.08)	10958.23 (1406.33)	11039.76 (1917.00)	9681.75 (1512.47)

Table S2: Investigators at each site

No.	Participating Sites	Investigators
01	Qilu Hospital of Shandong University	Ming Hou
02	Anhui Province Hospital	Changcheng Zheng
03	The First Affiliated Hospital of Anhui Medical University	Ruixiang Xia
04	The First Affiliated Hospital of Bengbu Medical College	Feng Zhang
07	The First Hospital of Changsha	Yongqing Cao
08	Heping Hospital Affiliated to Changzhi Medical College	Xuliang Shen
09	Fujian Medical University Union Hospital	Meijuan Huang
10	Hainan General Hospital	Hongxia Yao
11	Henan Cancer Hospital Affiliated Cancer Hospital of Zhengzhou University	Hu Zhou
12	The First Hospital of Jilin University	Sujun Gao
13	Affiliated Hospital of Jiangsu University	Xiaoming Fei
14	The Second Affiliated Hospital of Kunming Medical University	Zeping Zhou
16	Lanzhou University Second Hospital	Lingling Yue

17	The First Hospital of Lanzhou University	Yaming Xi
18	The First Affiliated Hospital of Nanchang University	Ruibin Huang
19	The Affiliated Hospital of Qingdao University	Wei Wang
20	Qingdao Central Hospital	Ling Wang
22	Shenzhen Second People’s Hospital	Xin Du
23	Suining Central Hospital	Xin Wang
24	Weihai Central Hospital	Binghua Wang
25	Weifang People’s Hospital	Xuehong Ran
26	Zhongnan Hospital of Wuhan University	Xuelan Zuo
27	The Central Hospital of Wuhan	Hongxiang Wang
28	The First Affiliated Hospital of Xinjiang Medical University	Xinhong Guo
29	Yichang Central People’s Hospital	Jingming Guo
30	The First People’s Hospital of Yunnan Province	Tonghua Yang
31	The First Affiliated Hospital Zhejiang University School of Medicine	Jie Jin
32	Xinqiao Hospital of Army Medical University	Xi Zhang

33	The First Hospital of China Medical University	Yan Li
34	Shengjing Hospital of China Medical University	Wei Yang
36	Affiliated Hospital of Zunyi Medical University	Zhong Yuan
40	Nanfang Hospital	Jing Sun
41	The Second Affiliated Hospital of Guangzhou Medical University	Xu Ye
43	Qinghai University Affiliated Hospital	Hui Geng
44	The Affiliated Hospital of Inner Mongolia Medical University	Da Gao
45	Affiliated Hospital of Chengde Medical University	Zhihua Zhang
46	Tianjin Medical University General Hospital	Rong Fu
47	Zhongda Hospital Southeast University	Zheng Ge
48	The Affiliated Huaian No.1 People’s Hospital of Nanjing Medical University	Chunling Wang
49	The First Affiliated Hospital of Zhejiang Univesity School of Medicine (Geriatrics Deparment)	Xiujin Ye

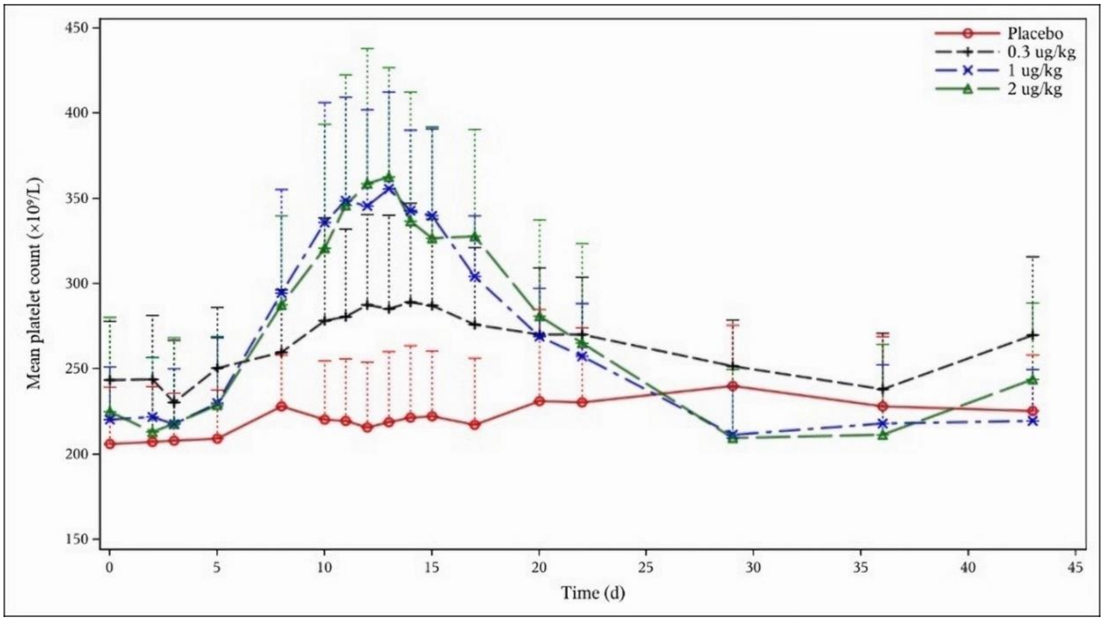

Figure S1: Mean platelet count-time profiles after a single dose of QL0911 or placebo in phase I study.

2A

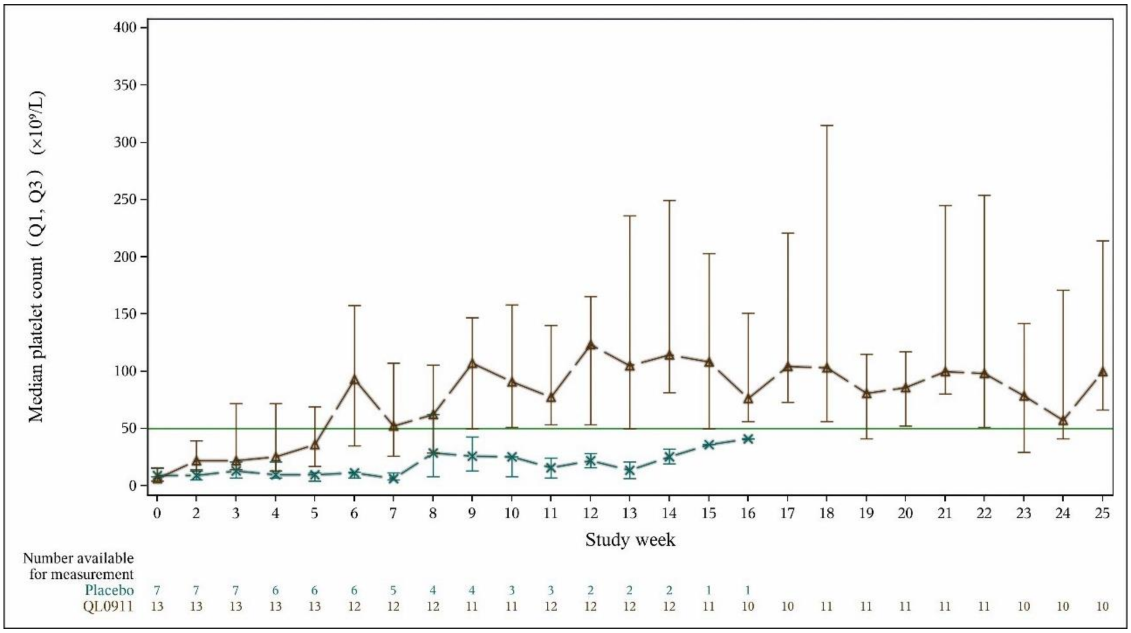

2B

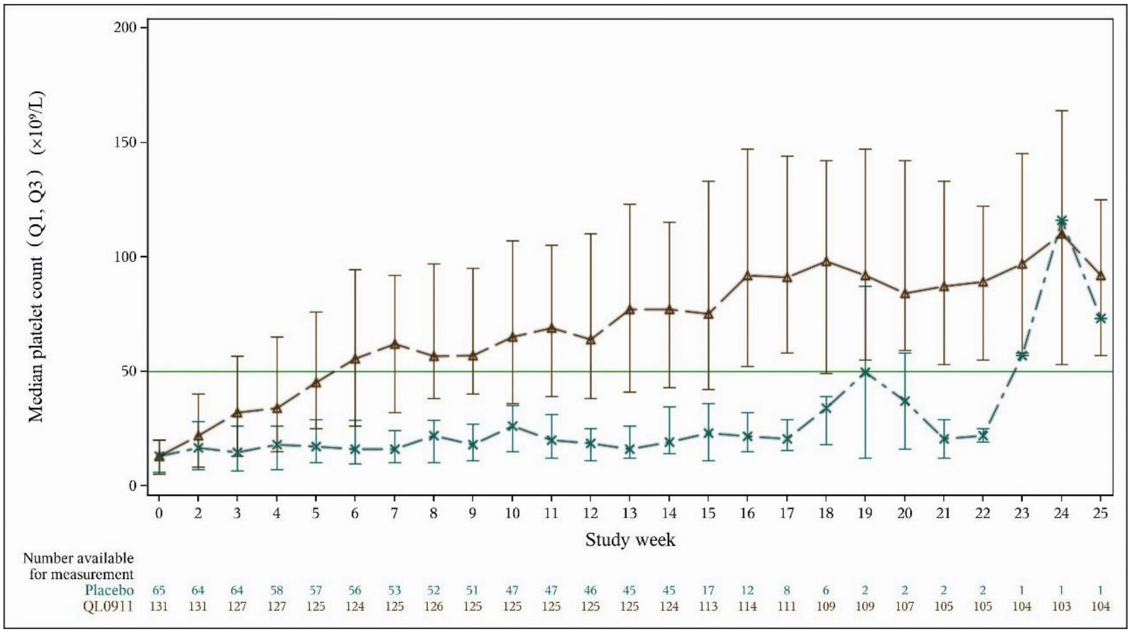
Median platelet count（Q1, Q3）(×10⁹/L)
Study week
Number available for measurement
Placebo 65 64 64 58 57 56 53 52 51 47 47 46 45 45 17 12 8 6 2 2 2 2 1 1 1
QL0911 131 131 127 127 125 124 125 126 125 125 125 125 125 124 113 114 111 109 109 107 105 105 104 103 104

2C

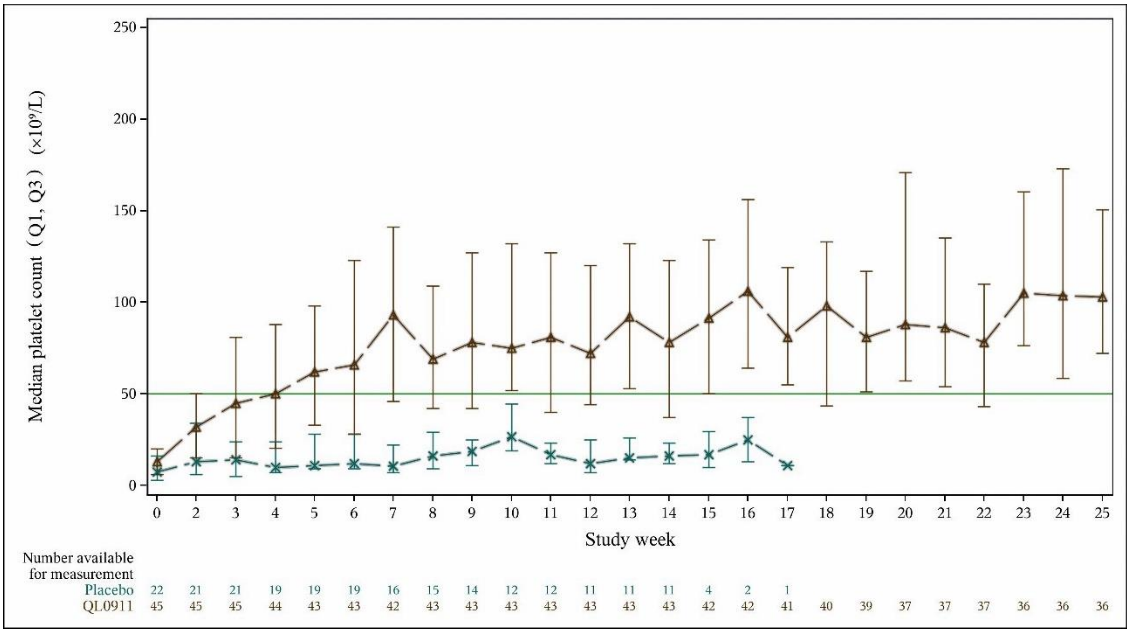
Median platelet count（Q1, Q3）(×10⁹/L)
Study week
Number available for measurement
Placebo 22 21 21 19 19 19 16 15 14 12 12 11 11 11 4 2 1
QL0911 45 45 45 44 43 43 42 43 43 43 43 43 43 43 42 42 41 40 39 37 37 37 36 36 36

2D

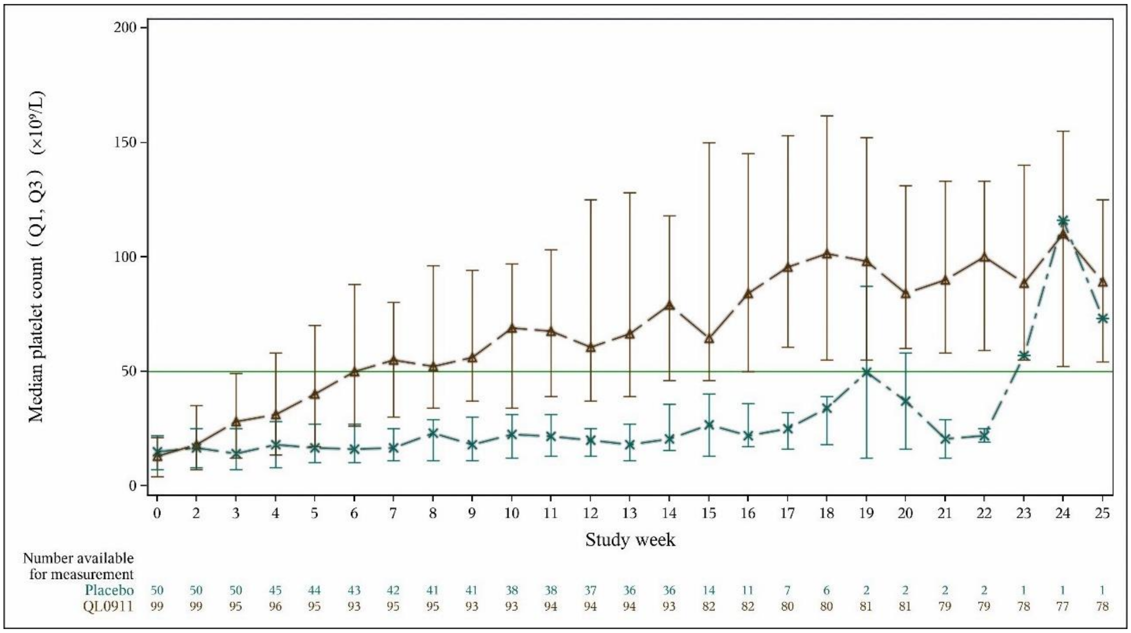

Figure S2: Median platelet counts at each weekly visit during the double-blind, 24-week treatment period in splenectomized (A) and non-splenectomized patients (B); in patients with baseline concomitant ITP treatment (C); and without baseline concomitant ITP treatment (D).

3A

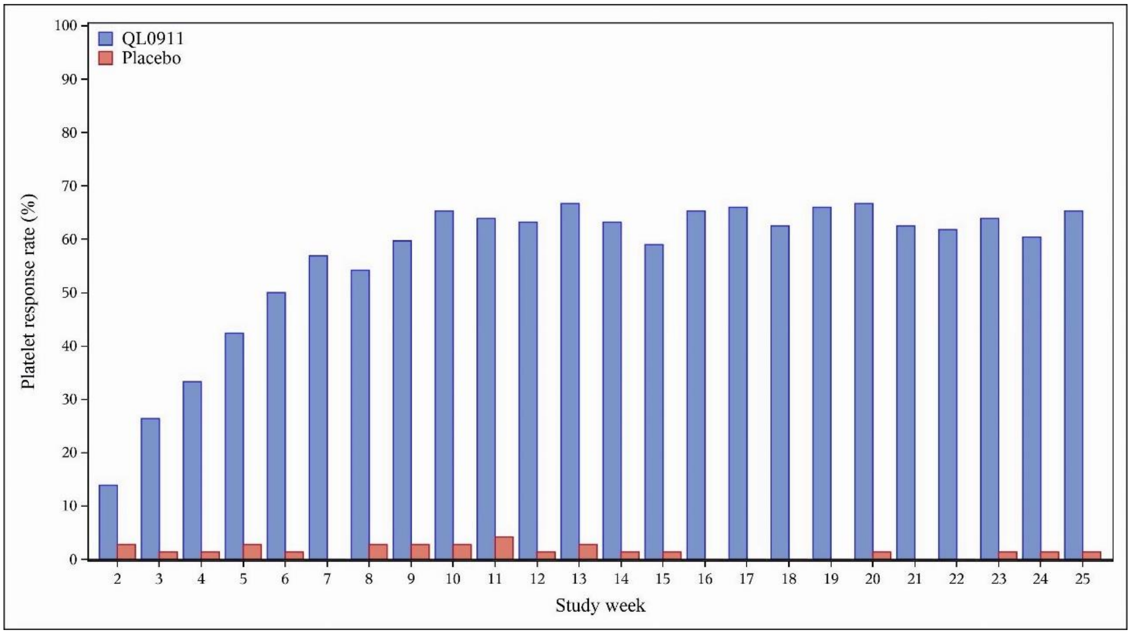

3B

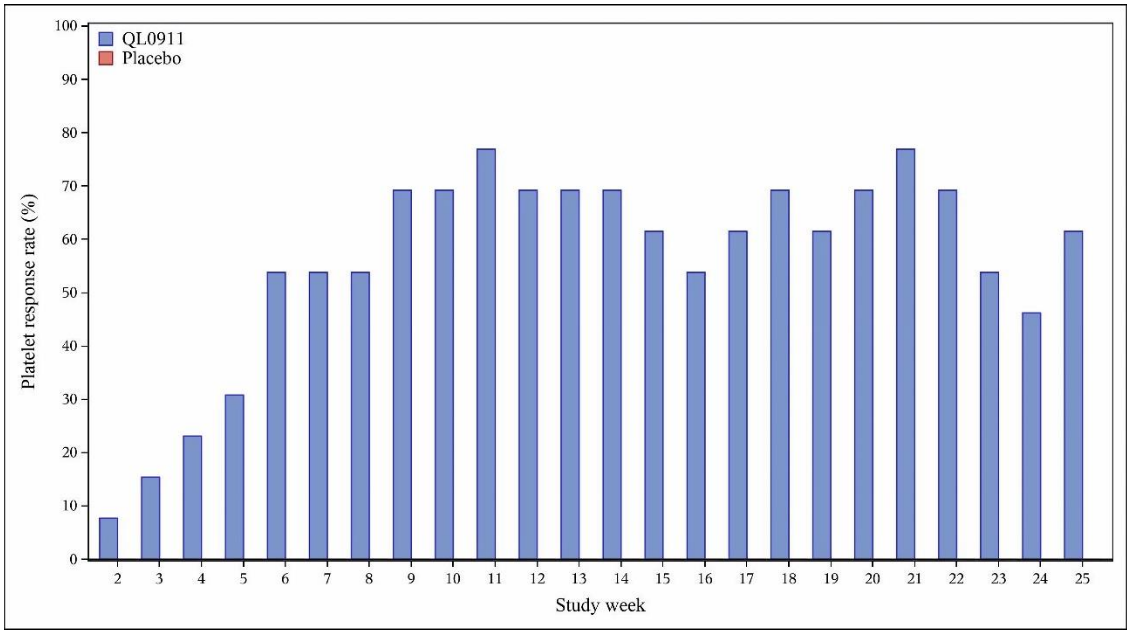
QL0911
Placebo
Platelet response rate (%)
Study week

3C

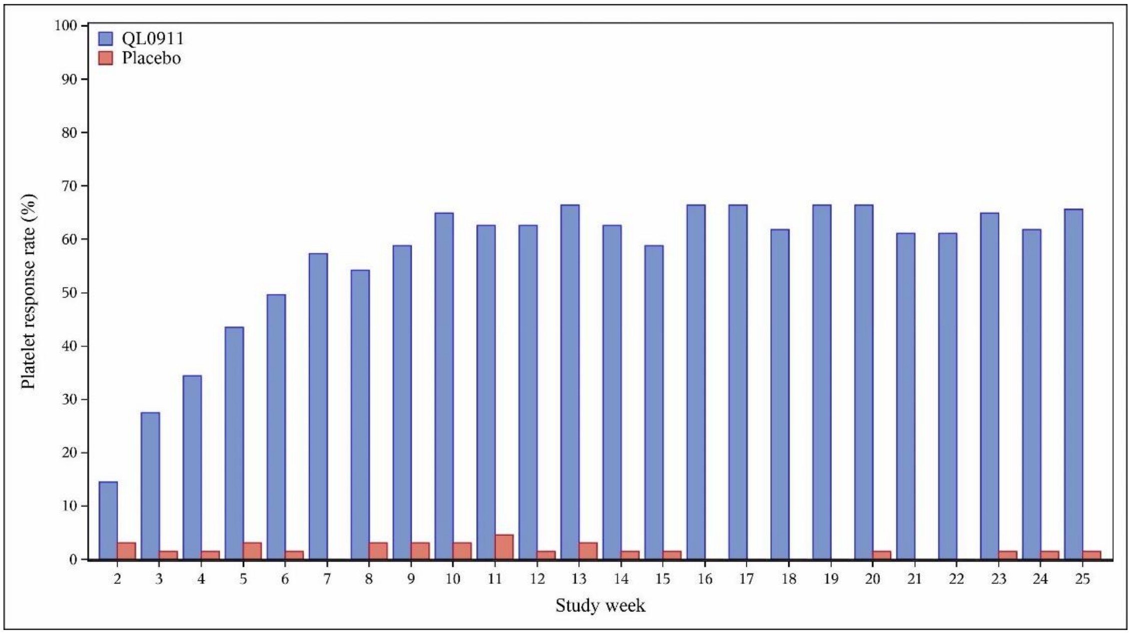
QL0911
Placebo
Platelet response rate (%)
Study week

3D

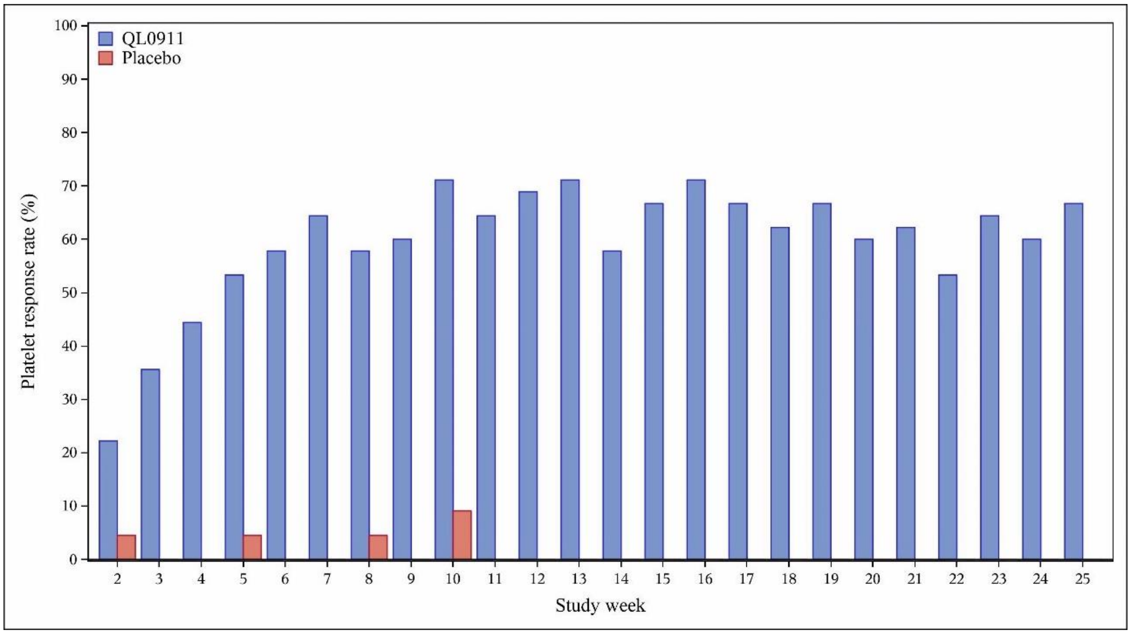

3E

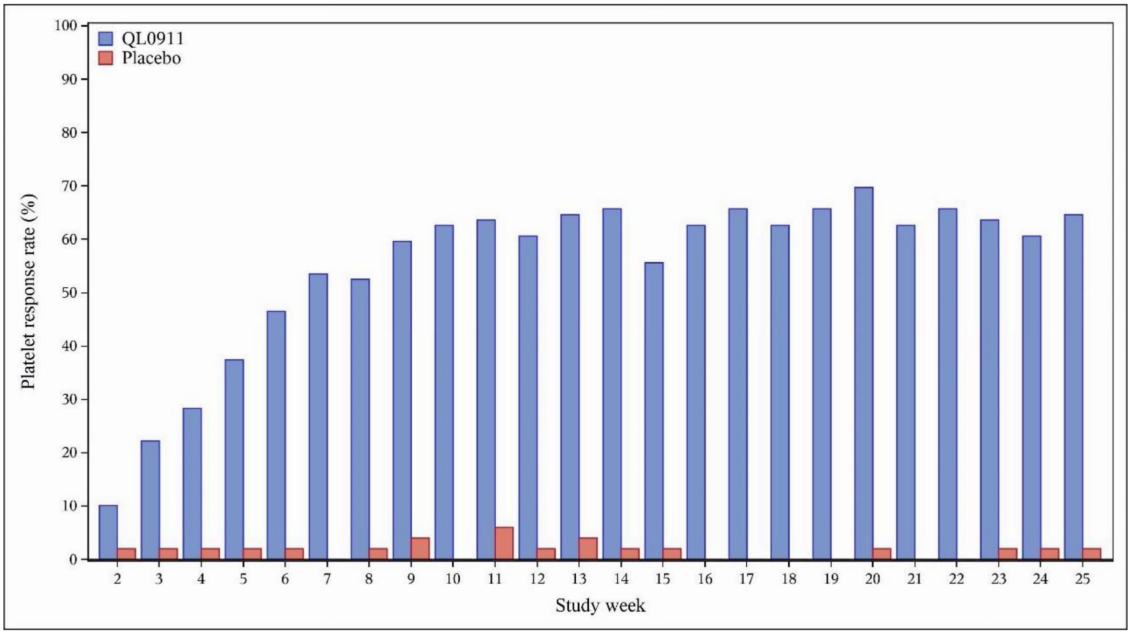

Figure S3: Platelet response rate in total population (A); in splenectomized or non-splenectomized patients (B and C), in patients with or without baseline concomitant ITP therapy (D and E) during the double-blind, 24-week treatment period.

4A

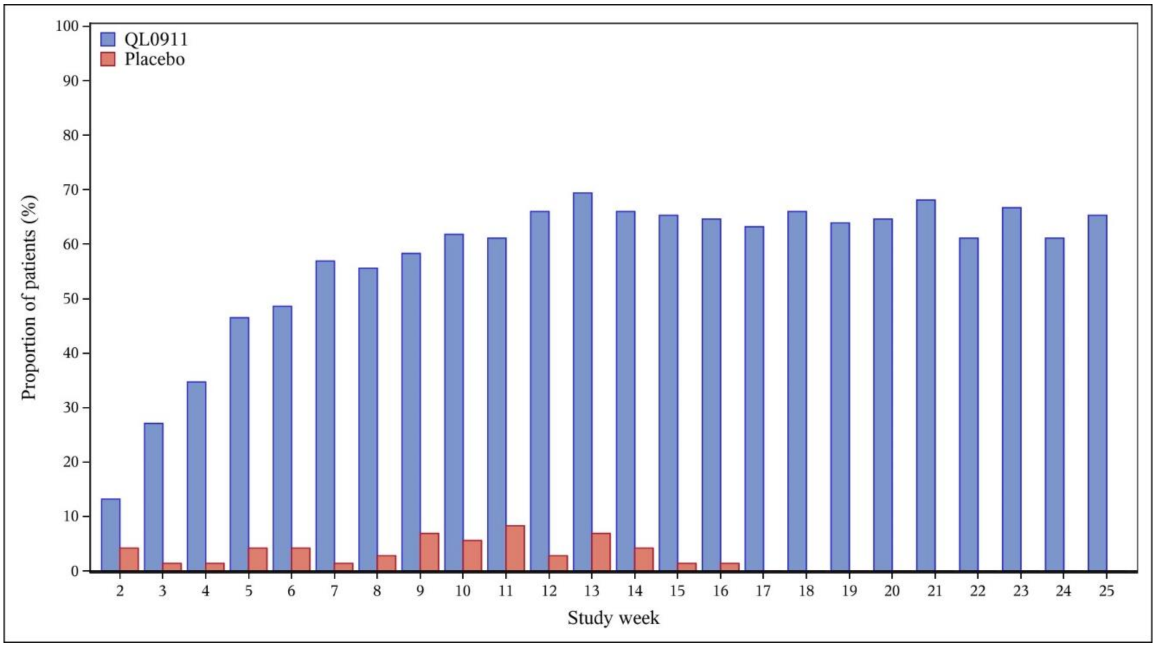
QL0911
Placebo
Proportion of patients (%)
Study week
0
10
20
30
40
50
60
70
80
90
100
2
3
4
5
6
7
8
9
10
11
12
13
14
15
16
17
18
19
20
21
22
23
24
25

4B

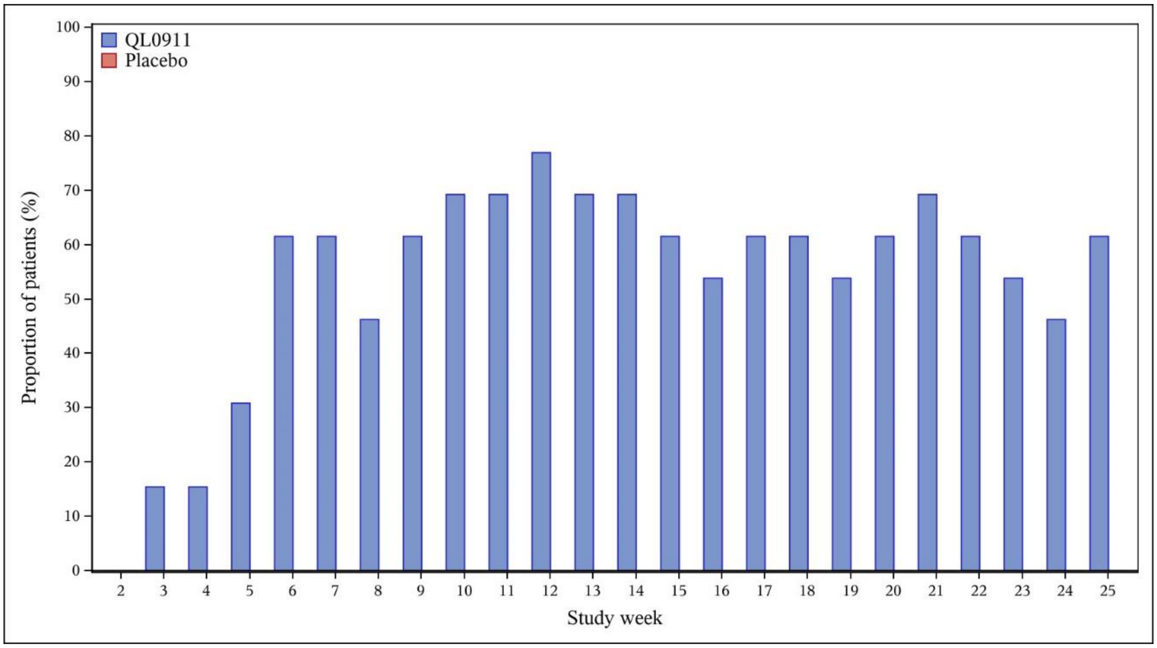
QL0911
Placebo
Proportion of patients (%)
Study week
0
10
20
30
40
50
60
70
80
90
100
2
3
4
5
6
7
8
9
10
11
12
13
14
15
16
17
18
19
20
21
22
23
24
25

4C

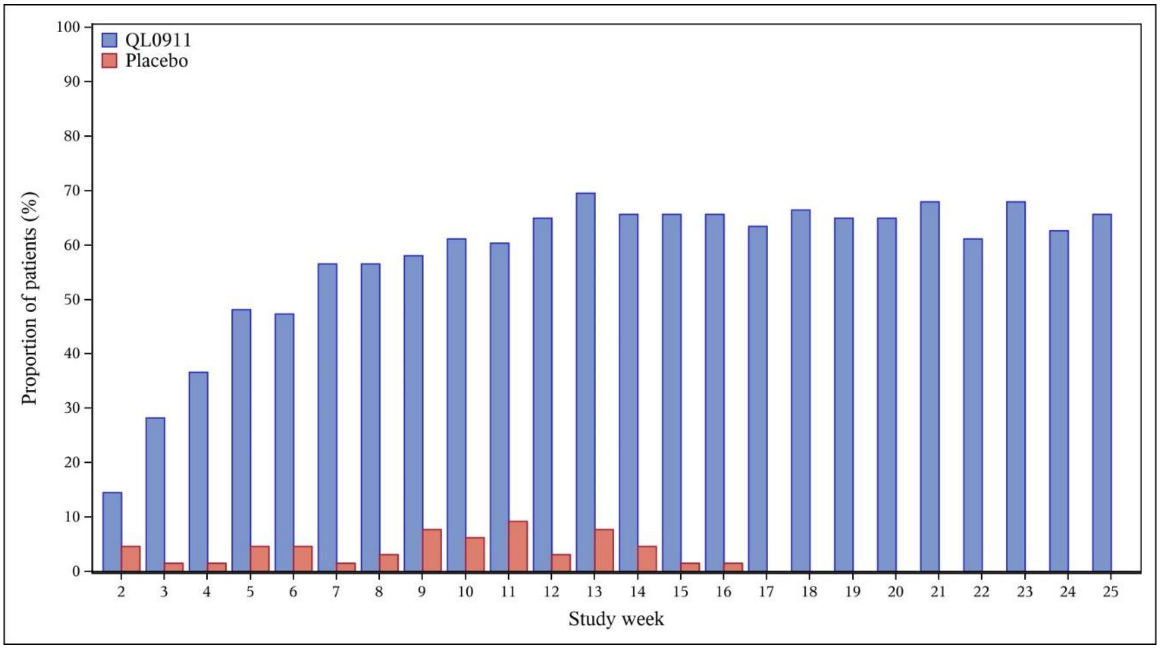
QL0911
Placebo
Proportion of patients (%)
100
90
80
70
60
50
40
30
20
10
0
2 3 4 5 6 7 8 9 10 11 12 13 14 15 16 17 18 19 20 21 22 23 24 25
Study week

4D

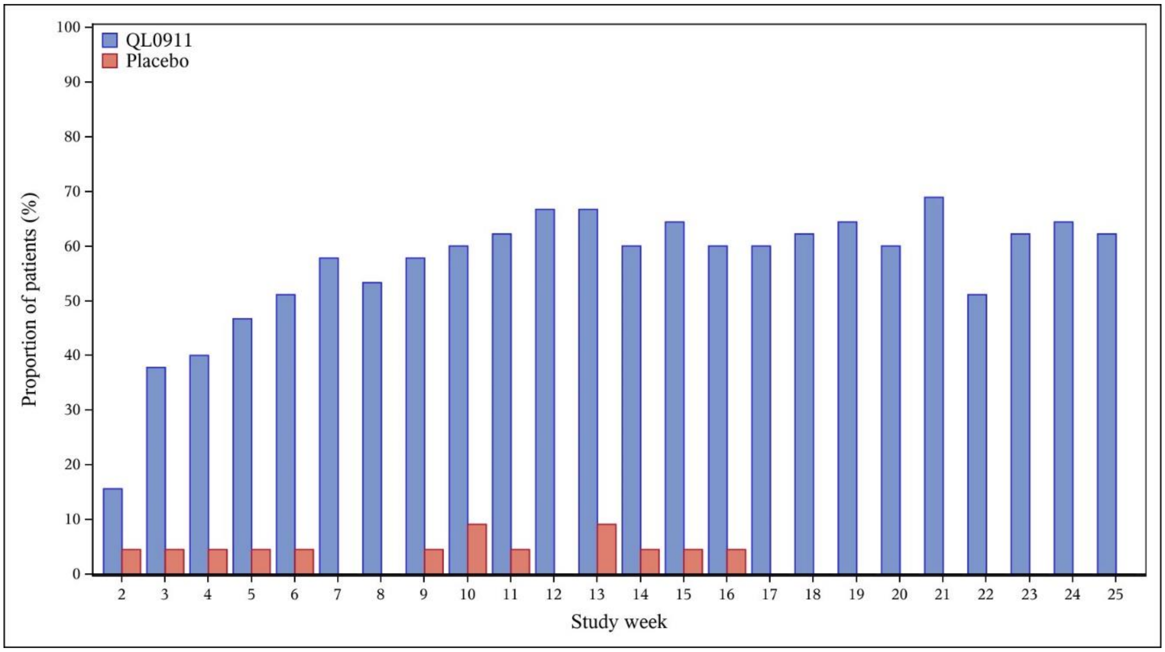
QL0911
Placebo
Proportion of patients (%)
100
90
80
70
60
50
40
30
20
10
0
2 3 4 5 6 7 8 9 10 11 12 13 14 15 16 17 18 19 20 21 22 23 24 25
Study week

4E

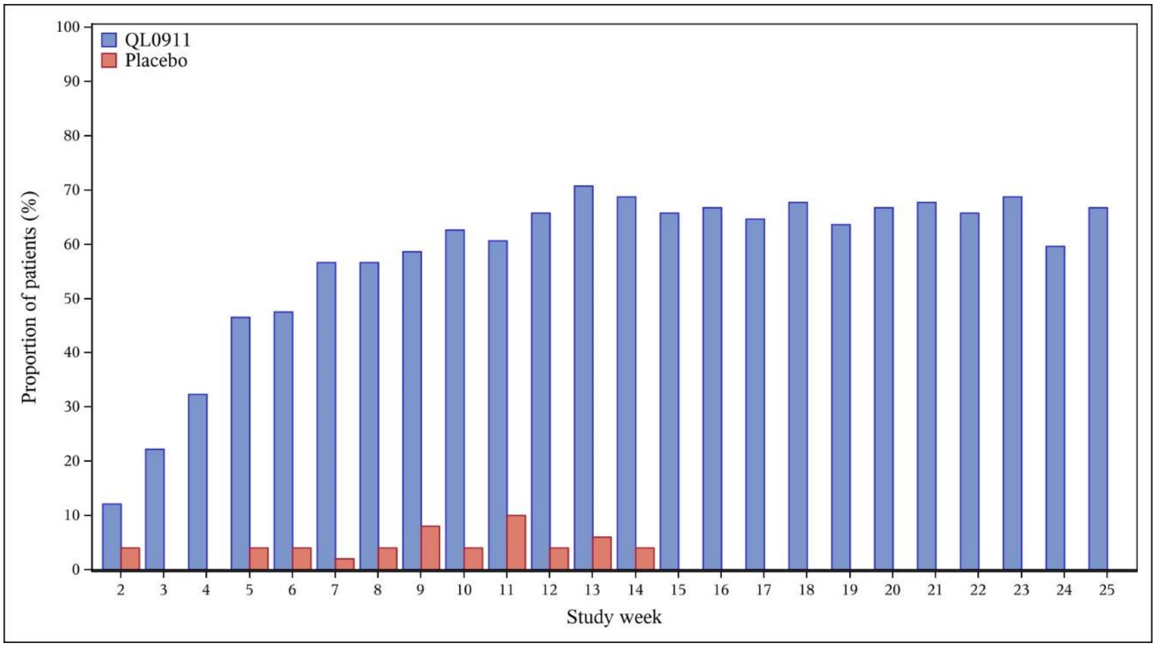

Figure S4: Proportion of patients with weekly platelet count ≥ 30 × 10^9/L and with a ≥ 2-fold increased platelet count from baseline and without bleeding events in total population (A), in splenectomized or non-splenectomized patients (B and C), in patients with or without baseline concomitant ITP therapy (D and E) during the double-blind, 24-week treatment period.

5A

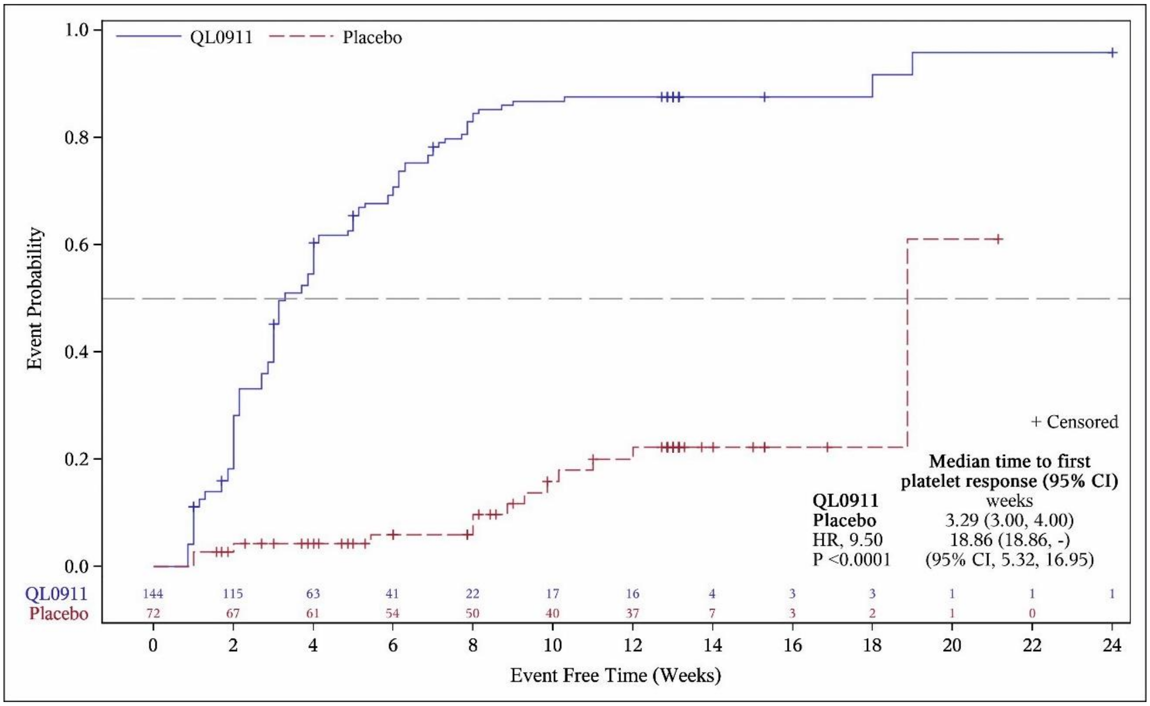

5B

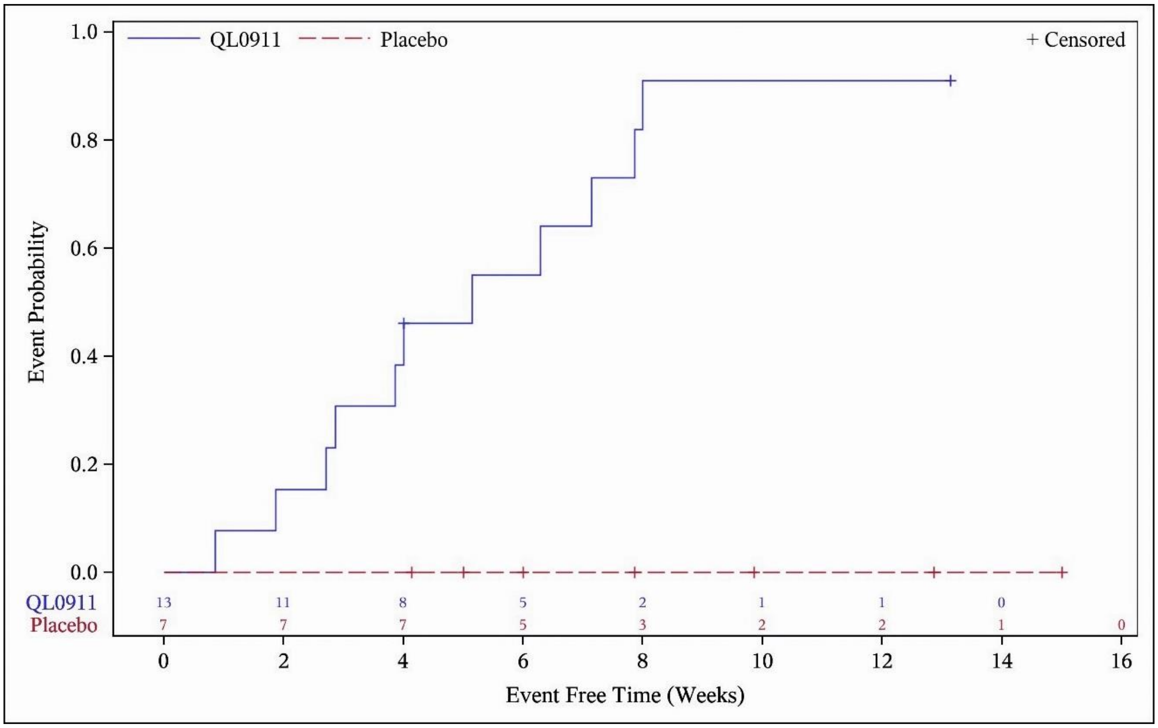
QL0911
Placebo
+ Censored
Event Probability
1.0
0.8
0.6
0.4
0.2
0.0
QL0911 13 11 8 5 2 1 1 0
Placebo 7 7 7 5 3 2 2 1 0
0 2 4 6 8 10 12 14 16
Event Free Time (Weeks)

5C

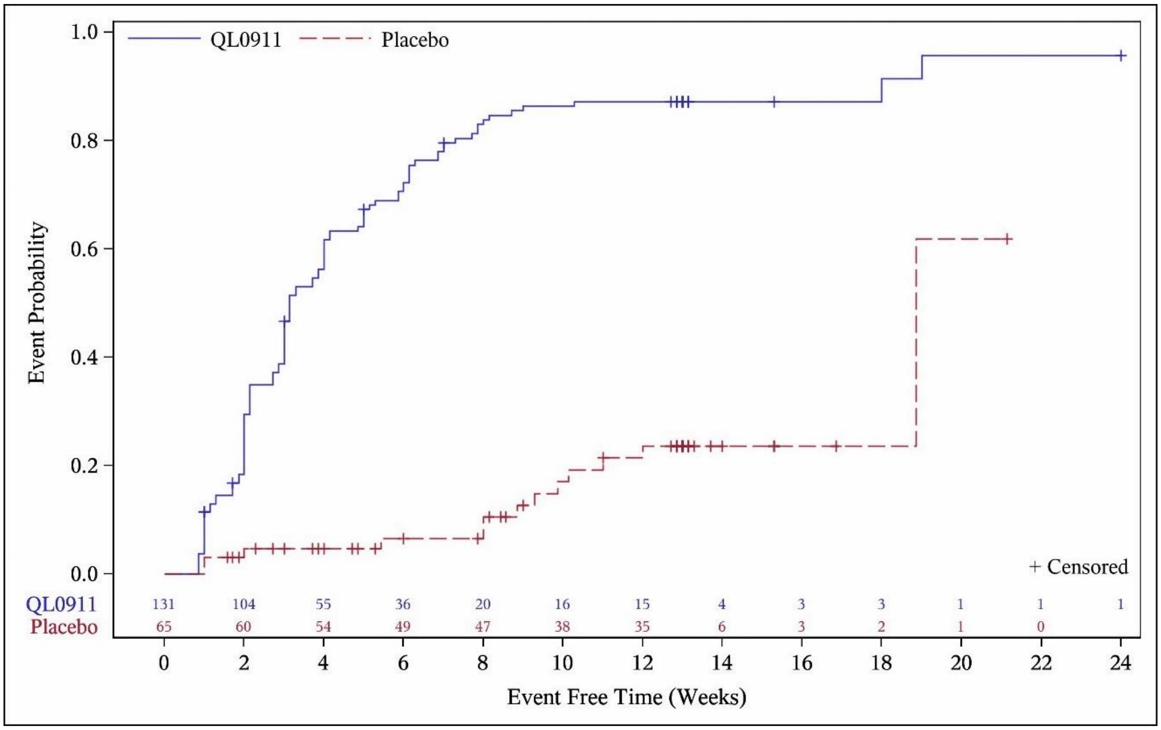
QL0911
Placebo
+ Censored
Event Probability
1.0
0.8
0.6
0.4
0.2
0.0
QL0911 131 104 55 36 20 16 15 4 3 3 1 1 1
Placebo 65 60 54 49 47 38 35 6 3 2 1 0
0 2 4 6 8 10 12 14 16 18 20 22 24
Event Free Time (Weeks)

5D

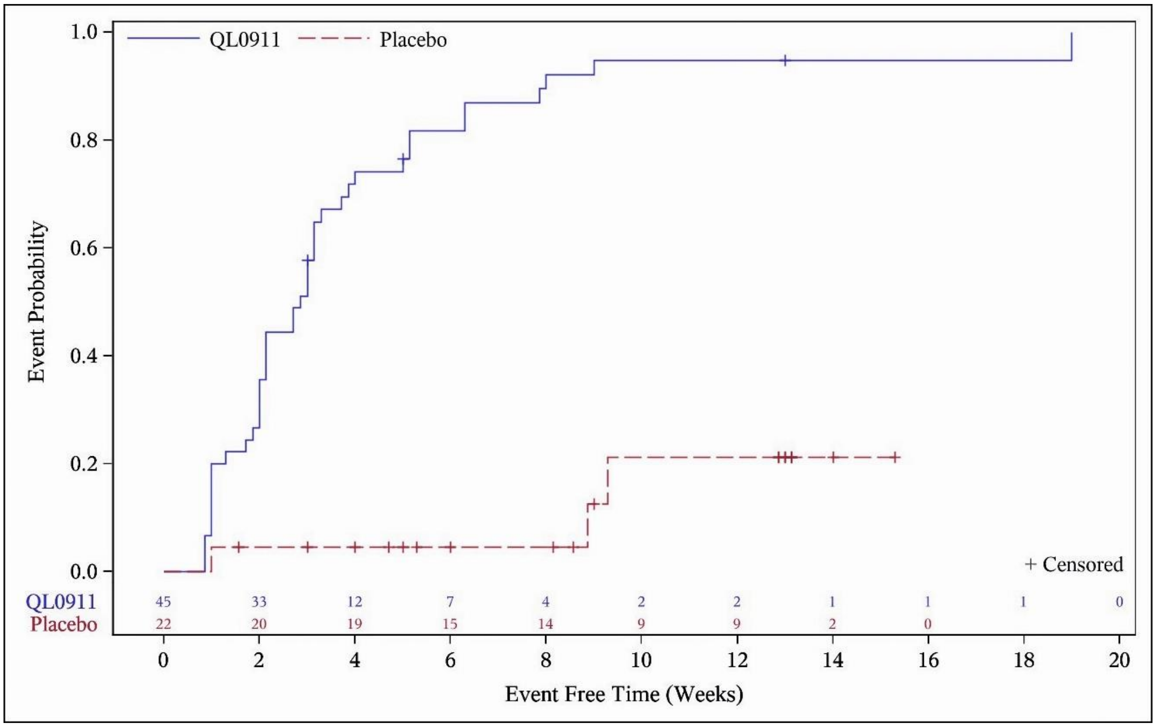

5E

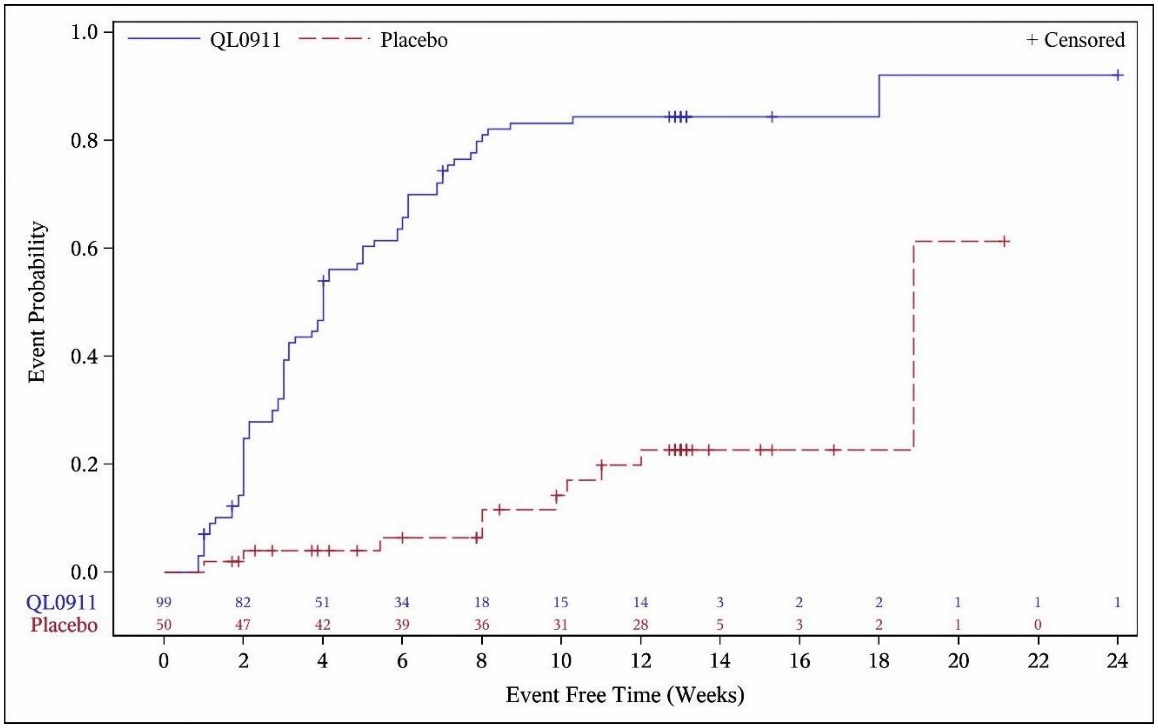

Figure S5: Kaplan-Meier curve of time to the first achieving platelet count ≥ 50 × 10^9/L in total population (A), in splenectomized or non-splenectomized patients (B and C), in patients with or without baseline concomitant ITP therapy (D and E) during the double-blind, 24-week treatment period.

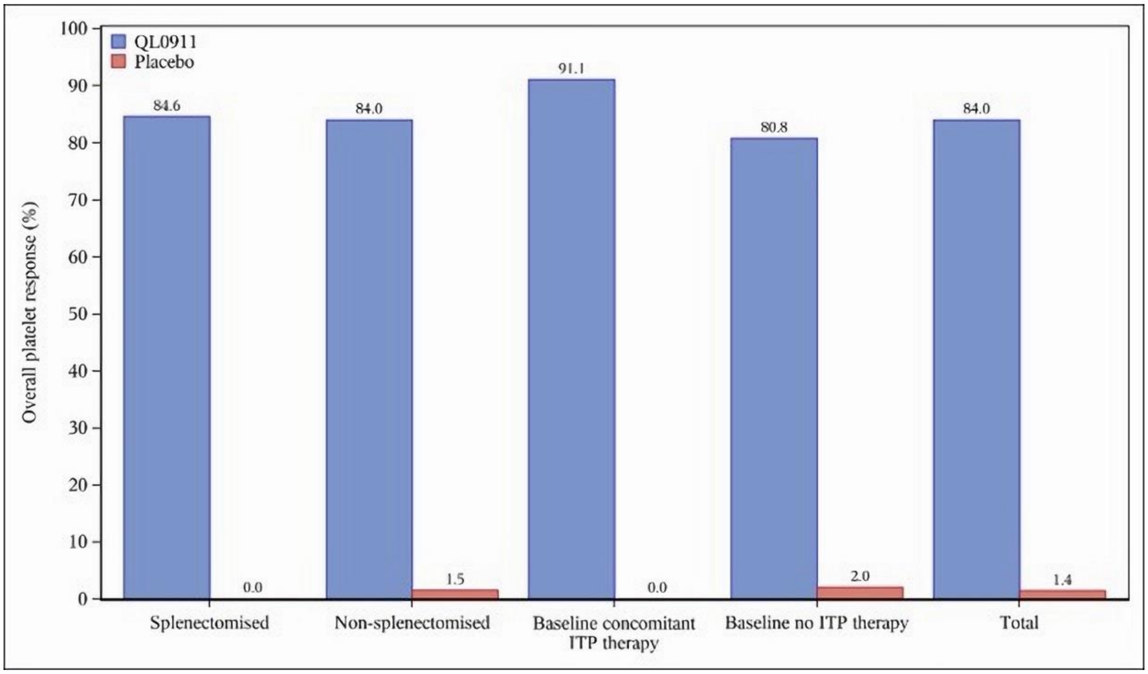

Figure S6: Proportion of patients with overall platelet response during the double-blind, 24-week treatment period. The overall platelet response was defined as either a durable response or at least four of the weekly platelet counts greater than 50×10^9/L at any time during the 24-week treatment period.
